# Electromagnetic fields act *via* activation of voltage-gated calcium channels to produce beneficial or adverse effects

**DOI:** 10.1111/jcmm.12088

**Published:** 2013-06-26

**Authors:** Martin L Pall

**Affiliations:** Professor Emeritus of Biochemistry and Basic Medical Sciences, Washington State UniversityPortland, OR, USA

**Keywords:** intracellular Ca^2+^, voltage-gated calcium channels, low frequency electromagnetic field exposure, nitric oxide, oxidative stress, calcium channel blockers

## Abstract

The direct targets of extremely low and microwave frequency range electromagnetic fields (EMFs) in producing non-thermal effects have not been clearly established. However, studies in the literature, reviewed here, provide substantial support for such direct targets. Twenty-three studies have shown that voltage-gated calcium channels (VGCCs) produce these and other EMF effects, such that the L-type or other VGCC blockers block or greatly lower diverse EMF effects. Furthermore, the voltage-gated properties of these channels may provide biophysically plausible mechanisms for EMF biological effects. Downstream responses of such EMF exposures may be mediated through Ca^2+^/calmodulin stimulation of nitric oxide synthesis. Potentially, physiological/therapeutic responses may be largely as a result of nitric oxide-cGMP-protein kinase G pathway stimulation. A well-studied example of such an apparent therapeutic response, EMF stimulation of bone growth, appears to work along this pathway. However, pathophysiological responses to EMFs may be as a result of nitric oxide-peroxynitrite-oxidative stress pathway of action. A single such well-documented example, EMF induction of DNA single-strand breaks in cells, as measured by alkaline comet assays, is reviewed here. Such single-strand breaks are known to be produced through the action of this pathway. Data on the mechanism of EMF induction of such breaks are limited; what data are available support this proposed mechanism. Other Ca^2+^-mediated regulatory changes, independent of nitric oxide, may also have roles. This article reviews, then, a substantially supported set of targets, VGCCs, whose stimulation produces non-thermal EMF responses by humans/higher animals with downstream effects involving Ca^2+^/calmodulin-dependent nitric oxide increases, which may explain therapeutic and pathophysiological effects.

- Introduction
- Possible modes of action following voltage-gated calcium channel stimulation
- Therapeutic bone-growth stimulation *via* Ca^2+^/nitric oxide/cGMP/protein kinase G
- Ca^2+^/nitric oxide/peroxynitrite and pathophysiological responses to EMF exposures: the example of single-strand DNA breaks
- Discussion and conclusions

## Introduction

An understanding of the complex biology of the effects of electromagnetic fields (EMFs) on human/higher animal biology inevitably must be derived from an understanding of the target or targets of such fields in the impacted cells and tissues. Despite this, no understanding has been forthcoming on what those targets are and how they may lead to the complex biological responses to EMFs composed of low-energy photons. The great puzzle, here, is that these EMFs are comprised of low-energy photons, those with insufficient energy to individually influence the chemistry of the cell, raising the question of how non-thermal effects of such EMFs can possibly occur. The author has found that there is a substantial literature possibly pointing to the direct targets of such EMFs and it is the goal of this study to review that evidence as well as review how those targets may lead to the complex biology of EMF exposure.

The role of increased intracellular Ca^2+^ following EMF exposure was already well documented more than 20 years ago, when Walleczek [1] reviewed the role of changes in calcium signalling that were produced in response EMF exposures. Other, more recent studies have confirmed the role of increased intracellular Ca^2+^ following EMF exposure, a few of which are discussed below. His review [1] included two studies [2, 3] that showed that the L-type voltage-gated channel blocker, verapamil could lower or block changes in response to EMFs. The properties of voltage-gated calcium channels (VGCCs) have been reviewed elsewhere [4]. Subsequently, extensive evidence has been published clearly showing that the EMF exposure can act to produce excessive activity of the VGCCs in many cell types [5–26] suggesting that these may be direct targets of EMF exposure. Many of these studies implicate specifically the L-type VGCCs such that various L-type calcium channel blockers can block responses to EMF exposure (Table 1). However, other studies have shown lowered responses produced by other types of calcium channel blockers including N-type, P/Q-type, and T-type blockers (Table 1), showing that other VGCCs may have important roles. Diverse responses to EMFs are reported to be blocked by such calcium channel blockers (Table 1), suggesting that most if not all EMF-mediated responses may be produced through VGCC stimulation. Voltage-gated calcium channels are essential to the responses produced by extremely low frequency (including 50/60 Hz) EMFs and also to microwave frequency range EMFs, nanosecond EMF pulses, and static electrical and magnetic fields (Table 1).

**Table 1:** EMF responses blocked or lowered by calcium channel blockers

Ref. no.	EMF type	Calcium channel	Cell type or organism	Response measured
2	Pulsed magnetic fields	L-type	Human lymphocytes	Cell proliferation; cytokine production
3	Static magnetic field (0.1 T)	L-type	Human polymorphonuclear leucocytes	Cell migration; degranulation
5	ELF	L-type	Rat chromaffin cells	Differentiation; catecholamine release
6	Electric field	L-type	Rat and mouse bone cells	Increased Ca^2+^, phospholipase A2, PGE2
7	50 Hz	L-type	Mytilus (mussel) immunocytes	Reduced shape change, cytotoxicity
8	50 Hz	L-type	AtT20 D16V, mouse pituitary corticotrope-derived	Ca^2+^ increase; cell morphology, premature differentiation
9	50 Hz	L-type	Neural stem/progenitor cells	*In vitro* differentiation, neurogenesis
10	Static magnetic field	L-type	Rat	Reduction in oedema formation
11	NMR	L-type	Tumour cells	Synergistic effect of EMF on anti-tumour drug toxicity
12	Static magnetic field	L-type	Myelomonocytic U937 cells	Ca^2+^ influx into cells and anti-apoptotic effects
13	60 Hz	L-type	Mouse	Hyperalgesic response to exposure
14	Single nanosecond electric pulse	L-type	Bovine chromaffin cells	Very rapid increase in intracellular Ca^2+^
15	Biphasic electric current	L-type	Human mesenchymal stromal cells	Osteoblast differentiation and cytokine production
16	DC & AC magnetic fields	L-type	β-cells of pancreas, patch clamped	Ca^2+^ flux into cells
17	50 Hz	L-type	Rat pituitary cells	Ca^2+^ flux into cells
18	50 Hz	L-type, N-type	Human neuroblastoma IMR32 and rat pituitary GH3 cells	Anti-apoptotic activity
19	Nanosecond pulse	L-type, N-type, P/Q-type	Bovine chromaffin cells	Ca^2+^ dynamics of cells
20	50 Hz	Not determined	Rat dorsal root ganglion cells	Firing frequency of cells
21	700–1100 MHz	N-type	Stem cell–derived neuronal cells	Ca^2+^ dynamics of cells
22	Very weak electrical fields	T-type	Sharks	Detection of very weak magnetic fields in the ocean
23	Short electric pulses	L-type	Human eye	Effect on electro-oculogram
24	Weak static magnetic field	L-type	Rabbit	Baroreflex sensitivity
25	Weak electric fields	T-type	Neutrophils	Electrical and ion dynamics
26	Static electric fields, ‘capacitive’	L-type	Bovine articular chondrocytes	Agrican & type II collagen expression; calcineurin and other Ca^2+^/calmodulin responses

EMF: electromagnetic field; ELF: extremely low frequency.

In a recent study, Pilla [27] showed that an increase in intracellular Ca^2+^ must have occurred almost immediately after EMF exposure, producing a Ca^2+^/calmodulin-dependent increase in nitric oxide occurring in less than 5 sec. Although Pilla [27] did not test whether VGCC stimulation was involved in his study, there are few alternatives that can produce such a rapid Ca^2+^ response, none of which has been implicated in EMF responses. Other studies, each involving VGCCs, summarized in Table 1, also showed rapid Ca^2+^ increases following EMF exposure [8, 16, 17, 19, 21]. The rapidity of these responses rule out many types of regulatory interactions as being involved in producing the increased VGCC activity following EMF exposure and suggests, therefore, that VGCC stimulation in the plasma membrane is directly produced by EMF exposure.

## Possible modes of action following VGCC stimulation

The increased intracellular Ca^2+^ produced by such VGCC activation may lead to multiple regulatory responses, including the increased nitric oxide levels produced through the action of the two Ca^2+^/calmodulin-dependent nitric oxide synthases, nNOS and eNOS. Increased nitric oxide levels typically act in a physiological context through increased synthesis of cGMP and subsequent activation of protein kinase G [28, 29]. In contrast, in most pathophysiological contexts, nitric oxide reacts with superoxide to form peroxynitrite, a potent non-radical oxidant [30, 31], which can produce radical products, including hydroxyl radical and NO_2_ radical [32].

## Therapeutic bone-growth stimulation *via* Ca^2+^/nitric oxide/cGMP/protein kinase G

An example of a therapeutic effect for bone repair of EMF exposure in various medical situations includes increasing osteoblast differentiation and maturation and has been reviewed repeatedly [33–44]. The effects of EMF exposure on bone cannot be challenged, although there is still considerable question about the best ways to apply this clinically [33–44]. Our focus, here, is to consider possible mechanisms of action. Multiple studies have implicated increased Ca^2+^ and nitric oxide in the EMF stimulation of bone growth [44–49]; three have also implicated increased cGMP and protein kinase G activity [46, 48, 49]. In addition, studies on other regulatory stimuli leading to increased bone growth have also implicated increased cGMP levels and protein kinase G in this response [50–56]. In summary, then, it can be seen from the above that there is a very well-documented action of EMFs in stimulating osteoblasts and bone growth. The available data, although limited, support the action of the main pathway involved in physiological responses to Ca^2+^ and nitric oxide, namely Ca^2+^/nitric oxide/cGMP/protein kinase G in producing such stimulation.

## Ca^2+^/nitric oxide/peroxynitrite and pathophysiological responses to EMF exposures: the example of single-strand DNA breaks

As was noted above, most of the pathophysiological effects of nitric oxide are mediated through peroxynitrite elevation and consequent oxidative stress. There are many reviews and other studies, implicating oxidative stress in generating pathophysiological effects of EMF exposure [see for example [57–64]]. In some of these studies, the rise in oxidative stress markers parallels the rise in nitric oxide, suggesting a peroxynitrite-mediated mechanism [64–67].

Peroxynitrite elevation is usually measured through a marker of peroxynitrite-mediated protein nitration, 3-nitrotyrosine (3-NT). There are four studies where 3-NT levels were measured before and after EMF exposure [66, 68–70]. Each of these studies provides some evidence supporting the view that EMF exposure increases levels of peroxynitrite and therefore 3-NT levels [66, 68–70]. Although these cannot be taken as definitive, when considered along with the evidence on oxidative stress and elevated nitric oxide production in response to EMF exposure, they strongly suggest a peroxynitrite-mediated mechanism of oxidative stress in response to EMFs.

Such a peroxynitrite-mediated mechanism may explain the many studies showing the single-stranded breaks in DNA, as shown by alkaline comet assays or the similar microgel electrophoresis assay, following EMF exposures in most such studies [71–89], but not in all [90–97]. Some of the factors that are reported to influence whether such DNA single-strand breaks are detected after EMF exposure include the type of cell studied [79, 86], dosage of EMF exposure [78] and the type of EMF exposure studied [73, 77]. Oxidative stress and free radicals have roles, both because there is a concomitant increase in oxidative stress and because antioxidants have been shown to greatly lower the generation of DNA single-strand breaks following EMF exposure [72, 75, 81, 82] as has also been shown for peroxynitrite-mediated DNA breaks produced under other conditions. It has also been shown that one can block the generation of DNA single-strand breaks with a nitric oxide synthase inhibitors [82].

Peroxynitrite has been shown to produce single-strand DNA breaks [98–100], a process that is inhibited by many but not all antioxidants [99, 100]. It can be seen from this that the data on generation of single-strand DNA breaks, although quite limited, support a mechanism involving nitric oxide/peroxynitrite/free radical (oxidative stress). Although the data on the possible role of peroxynitrite in EMF-induced DNA single-strand breaks are limited, what data are available supports such a peroxynitrite role.

## Discussion and conclusions

How do EMFs composed of low-energy photons produce non-thermal biological changes, both pathophysiological and, in some cases, potentially therapeutic, in humans and higher animals? It may be surprising that the answer to this question has been hiding in plain sight in the scientific literature. However, in this era of highly focused and highly specialized science, few of us have the time to read the relevant literature, let alone organize the information found within it in useful and critical ways.

This study shows that:

1. Twenty-three different studies have found that such EMF exposures act *via* activation of VGCCs, such that VGCC channel blockers can prevent responses to such exposures (Table 1). Most of the studies implicate L-type VGCCs in these responses, but there are also other studies implicating three other classes of VGCCs.
2. Both extremely low frequency fields, including 50/60 cycle exposures, and microwave EMF range exposures act *via* activation of VGCCs. So do static electric fields, static magnetic fields and nanosecond pulses.
3. Voltage-gated calcium channel stimulation leads to increased intracellular Ca^2+^, which can act in turn to stimulate the two calcium/calmodulin-dependent nitric oxide synthases and increase nitric oxide. It is suggested here that nitric oxide may act in therapeutic/potentially therapeutic EMF responses *via* its main physiological pathway, stimulating cGMP and protein kinase G. It is also suggested that nitric oxide may act in pathophysiological responses to EMF exposure, by acting as a precursor of peroxynitrite, producing both oxidative stress and free radical breakdown products.
4. The interpretation in three above is supported by two specific well-documented examples of EMF effects. Electromagnetic fields stimulation of bone growth, modulated through EMF stimulation of osteoblasts, appears to involve an elevation/nitric oxide/protein kinase G pathway. In contrast to that, it seems likely that the EMF induction of single-stranded DNA breaks involves a Ca^2+^/elevation/nitric oxide/peroxynitrite/free radical (oxidative stress) pathway.

It may be asked why we have evidence for involvement of VGCCs in response to EMF exposure, but no similar evidence for involvement of voltage-gated sodium channels? Perhaps, the reason is that there are many important biological effects produced in increased intracellular Ca^2+^, including but not limited to nitric oxide elevation, but much fewer are produced by elevated Na^+^.

The possible role of peroxynitrite as opposed to protein kinase G in producing pathophysiological responses to EMF exposure raises the question of whether there are practical approaches to avoiding such responses? Typically peroxynitrite levels can be highly elevated when both of its precursors, nitric oxide and superoxide, are high. Consequently, agents that lower nitric oxide synthase activity and agents that raise superoxide dismutases (SODs, the enzymes that degrade superoxide) such as phenolics and other Nrf2 activators that induce SOD activity [101], as well as calcium channel blockers may be useful. Having said that, this is a complex area, where other approaches should be considered, as well.

Although the various EMF exposures as well as static electrical field exposures can act to change the electrical voltage-gradient across the plasma membrane and may, therefore, be expected to stimulate VGCCs through their voltage-gated properties, it may be surprising that static magnetic fields also act to activate VGCCs because static magnetic fields do not induce electrical changes on static objects. However, cells are far from static. Such phenomena as cell ruffling [102],[103] may be relevant, where thin cytoplasmic sheets bounded on both sides by plasma membrane move rapidly. Such rapid movement of the electrically conducting cytoplasm, may be expected to influence the electrical charge across the plasma membrane, thus potentially stimulating the VGCCs.

Earlier modelling of electrical effects across plasma membranes of EMF exposures suggested that such electrical effects were likely to be too small to explain EMF effects at levels reported to produce biological changes (see, for example [22]). However, more recent and presumably more biologically plausible modelling have suggested that such electrical effects may be much more substantial [104–109] and may, therefore, act to directly stimulate VGCCs.

Direct stimulation of VGCCs by partial depolarization across the plasma membrane is suggested by the following observations discussed in this review:

1. The very rapid, almost instantaneous increase in intracellular Ca^2+^ found in some studies following EMF exposure [8, 16, 17, 19, 21, 27]. The rapidity here means that most, if not all indirect, regulatory effects can be ruled out.
2. The fact that not just L-type, but three additional classes of VGCCs are implicated in generating biological responses to EMF exposure (Table 1), suggesting that their voltage-gated properties may be a key feature in their ability to respond to EMFs.
3. Most, if not all, EMF effects are blocked by VGCC channel blockers (Table 1).
4. Modelling of EMF effects on living cells suggests that plasma membrane voltage changes may have key roles in such effects [104–109]. Saunders and Jefferys stated [110] that ‘It is well established that electric fields … or exposure to low frequency magnetic fields, will, if of sufficient magnitude, excite nerve tissue through their interactions with … voltage gated ion channels’. They further state [110] that this is achieved by direct effects on the electric dipole voltage sensor within the ion channel.

One question that is not answered by any of the available data is whether what is known as ‘dirty electricity’ [111–113], generated by rapid, in many cases, square wave transients in EMF exposure, also acts by stimulating VGCCs. Such dirty electricity is inherent in any digital technology because digital technology is based on the use of such square wave transients and it may, therefore, be of special concern in this digital era, but there have been no tests of such dirty electricity that determine whether VGCCs have roles in response to such fields, to my knowledge. The nanosecond pulses, which are essentially very brief, but high-intensity dirty electricity do act, at least in part, *via* VGCC stimulation (Table 1), suggesting that dirty electricity may do likewise. Clearly, we need direct study of this question.

The only detailed alternative to the mechanism of non-thermal EMF effects discussed here, to my knowledge, is the hypothesis of Friedman *et al*. [114] and supported by Desai *et al*. [115] where the apparent initial response to EMF exposure was proposed to be NADH oxidase activation, leading to oxidative stress and downstream regulatory effects. Although they provide some correlative evidence for a possible role of NADH oxidase [114], the only causal evidence is based on a presumed specific inhibitor of NADH oxidase, diphenyleneiodonium (DPI). However, DPI has been shown to be a non-specific cation channel blocker [116], clearly showing a lack of such specificity and suggesting that it may act, in part, as a VGCC blocker. Consequently, a causal role for NADH oxidase in responses to EMF exposure must be considered to be undocumented.

In summary, the non-thermal actions of EMFs composed of low-energy photons have been a great puzzle, because such photons are insufficiently energetic to directly influence the chemistry of cells. The current review provides support for a pathway of the biological action of ultralow frequency and microwave EMFs, nanosecond pulses and static electrical or magnetic fields: EMF activation of VGCCs leads to rapid elevation of intracellular Ca^2+^, nitric oxide and in some cases at least, peroxynitrite. Potentially therapeutic effects may be mediated through the Ca^2+^/nitric oxide/cGMP/protein kinase G pathway. Pathophysiological effects may be mediated through the Ca^2+^/nitric oxide/peroxynitrite pathway. Other Ca^2+^-mediated effects may have roles as well, as suggested by Xu *et al*. [26].
